# A qualitative study on the attitudes of women politicians toward their roles in politics: a case of Northern Cyprus

**DOI:** 10.3389/fpsyg.2023.1304905

**Published:** 2024-01-08

**Authors:** Niyper Hayal Artaç, Ebru Oğurlu

**Affiliations:** ^1^Department of Political Science and Public Administration, Faculty of Economics and Administrative Sciences, European University of Lefke, Lefke, Türkiye; ^2^Department of International Relations, Faculty of Economics and Administrative Sciences, European University of Lefke, Lefke, Türkiye

**Keywords:** barriers to political participation, feminism, leadership, political participation, female MPs

## Abstract

**Introduction:**

Politics is a mechanism of cooperation for the common interest of society. In this mechanism, each individual is expected to participate equally in the leadership and decision-making mechanisms. Women's participation in politics is essential for the spread of good governance and democracy. Globally, political participation is disaggregated by gender, with men's participation greater than women's. A lower representation of women in politics is also observed in Northern Cyprus. The attitudes or views of society and politicians may determine the political participation of women in leadership positions. This study examines the obstacles and determining factors that make it difficult for women to reach leadership positions even though their political role is increasing.

**Methods:**

Semi-structured interviews were conducted with 21 female participants for this research. Eleven of them are female ministers in parliament and represent the three main political parties in the country. The remaining 10 female participants are also members of the central executive body of the three parties and the National Assembly.

**Results and discussion:**

Women politicians believe that problems related to gender equality in politics prevent women from entering active politics and rising to leadership positions. Although there was no gender discrimination in Northern Cyprus means that women have gained equal rights with men in many areas, it was concluded that the traditional political culture keeps women out of politics.

## 1 Introduction

It is well-known that gender discrimination is one of the most important debates in almost every culture. Depending on ideology, region, and period, women have limited rights, less experience in public life, and limited access to positions of power (Masad, 2020). The truth is still that although women are recognized as equal to men in many areas, they have not reached the same authoritative status as men in politics (Schrupp, 2017). However, the free and equal participation of all elements that make up society in the administrative process and their representation through the protection of their interests are among the most important concerns of a democratic society. It is one of the main elements of democracy that women have the same place as men in every aspect of political life, especially in the decision-making mechanisms (Kenworthy and Malami, 1999). When political participation is broken down by gender, it is found that women's participation is lower than men's everywhere in the world. Several factors prevent women from participating in politics. In Northern Cyprus, some attitudes are consistent with this situation. Political leaders and members of society generally have biased views about how well women can participate in politics and govern the country. This originated from the attained roles for women in their society due to patriarchal structures in society and male-dominated politics (Hadjipavlou and Mertan, 2010). This study was conducted using a semi-structured interview structure with women politicians in the Republican Assembly of Northern Cyprus, the party councils, and central administrative councils of the three largest political parties in Northern Cyprus: Cumhuriyetçi Türk Partisi (CTP), Ulusal Birlik Partisi (UBP) and Halkin Partisi (HP). This research addresses the difficulties women politicians face in entering political life and the impact of gender inequality on their political life.

## 2 Literature review

### 2.1 Feminism

In this article, it is highlighted that it is usually difficult for women to rise to leadership positions even though they are active in political life by understanding the unequal gender dynamics through feminist theory. Feminism focuses on promoting women's position in society in terms of politics, economics, or legal rights (Tong, 2009). The important feminist theorists Bell Hooks and Estelle Freedman fought against the traditional norms and values as well as the sexist policies of male supremacy. They defined feminism as a movement to end gender inequality and devised solutions to women's problems. They argued that men and women are inherently equal and that both genders should work together to end gender discrimination (Hooks, 2000; Freedman, 2002). Feminism aims to protect women from oppression, exploitation, and discrimination regardless of race, language, religion, and nationality. In terms of gender, the distinction between public and private has emerged according to physiological characteristics. The core of this distinction is that women become pregnant, give birth to children, and care for and raise them. Men, on the other hand, use more physical strength to perform tasks that require power and endurance. Thus, the men became hunters, shepherds, or warriors; the women stayed at home, took care of the children, and did the housework. Over time, this division of roles has extended to all aspects of life. This is because while men are generally brave, strong, and independent, women are seen as passive, quiet, and caring (Vatandaş, 2007).

### 2.2 Differentiation in the role of women in public-private spheres

Feminism is generally considered to be an approach that opposes the prevailing patriarchal order in society and fights for women's rights to eliminate the problems women face in the private or public spheres (Kramer et al., 2019). This is because women's actions or experiences in the public sphere-work spheres/politics-are not seen as valuable to policymakers because of their status in the private sphere–domestic responsibilities. The dilemma between public and private spheres reinforces gender inequality and results in the man who owns the public sphere dominating the women who are in the private sphere. Female representation, which came to the fore in the first wave of feminism, is an important touchstone. Under the influence of this wind, women in many countries have gained legal advantages such as the right to vote, the right to be elected, the right to education, and the right to work. These gains are important, but insufficient based on traditional relationships. Even though egalitarian laws have been enacted today, women's political participation has not yet reached the same level as men's (Wischermann, 2004).

Understanding the concept of leadership is also important for this study because it primarily examines the reasons why women in Northern Cyprus do not achieve the desired level of leadership. Although women and men have many of the same rights in society, they do not have the same power as men in the political system of society (Duner, 1999). Although women have participated in the Republican Assembly of Northern Cyprus, they have made little progress in gaining real political power and leadership positions. For this reason, in light of feminist theory, women throughout the world are kept in the private sphere and cannot benefit from the opportunities that the public sphere offers (Holmes, 2022). When political participation is broken down by gender, it becomes clear that women's participation is lower than men's everywhere in the world. In democracies, individuals with equal rights are expected to participate equally in administrative and decision-making mechanisms (Hunt, 2007). In the context of equality, representation, and participation, women are found to be a more disadvantaged group than men around the world (De Nicola, 2017). However, in various societies, political leadership positions are assumed to be suited for men. By recognizing gender as an issue, the aim is to reduce the prejudice about women's “unsuitability” for leadership roles. Women have struggled to attain leadership positions in positions of influence. According to feminist theory, gender equality should be achieved by society to influence all institutions. Leaders not only influence other people but at the same time help shape the entire society and institutions. They also promote gender equality when they perform their duties (Chin et al., 2007). Therefore, strong leadership can contribute to the success of government and weak leadership can lead to failures in government. Leadership is a process of agreeing on what to do and how to do it, and encouraging individual and collective efforts to achieve common goals (Yukl, 2006).

### 2.3 Women's representation in politics

According to the concept of leadership, leaders can inspire citizens in their societies and shape the political structure. Leaders should be confident, willing to make difficult decisions, and compassionate toward those in need. Women's equal representation and engagement in politics and public life are critical components of a democracy. A more resilient, stronger, and inclusive world can result from empowering women as agents of change and decision-makers in the processes that shape their lives. Therefore, women's effective political participation is critical to contributing to improved human rights and sustainable development. For peace, democracy, and equality, women's perspectives and experiences must be considered on an equal footing with men's at all stages of the decision-making process (Mindzie, 2015). More women in political leadership positions benefit both women and society as a whole. Progress in policy areas is essential for economic growth and development when there are more women in political leadership positions. The case for women's empowerment is unassailable even without these findings. To address the problem of women's participation in political life, the “quota system” has been developed since the late 1980s (Galligan, 2006). Several governments around the world have afforded to increase women's political participation in policy-making mechanisms through legislative measures. Gender quota is a way to increase women's political participation by having a certain number of women in the decision-making mechanisms of political parties, local councils, and parliaments (Hughes et al., 2019). Not only do quotas promote demand for women's political participation, but they can also lead to changes in traditional norms, cultures, and behaviors in legislative assemblies and political parties (O'Brien and Rickne, 2016). These changes in political culture can reduce prejudice against women leaders, as people generally have a distrust of women's abilities in politics (Franceschet et al., 2012).

### 2.4 Historical overview of women's political rights in Northern Cyprus

The social structure of the Turkish Cypriot community is constantly and rapidly changing. Turkish Cypriot women are influenced by both Eastern and Western cultures (Mallinson, 2009). Women in Cyprus received the right to vote and stand for election many years after women in Turkey on December 5, 1934. The Turkish Family Law and the Turkish Family Court Law were considered the first steps toward equality for the Turkish Cypriot community. On May 28, 1951, the Turkish Family Law (Chapter 339) and the Turkish Family Court Law (Chapter 338) were passed in the Turkish Cypriot community and marked the beginning of the movement toward equality (Solsten, 1993). Following this first step, women received the right to equal education in 1952 and the right to vote and hold elective office in 1960, after the establishment of the Republic of Cyprus (Lisaniler, 2006). With the proclamation of the Republic of Cyprus (RoC) in 1960, women were given the right to vote and hold elective office, received education, and established organizations to protect women's rights. These developments accelerated the fact that women were more visible in the public sphere than in the private sphere (Mertan, 2000). The Convention on the Elimination of All Forms of Discrimination against Women (CEDAW) was declared on February 7, 1996 by the Republican Assembly of Northern Cyprus as a fundamental human rights convention and regulation for the protection of women's rights at the United Nations level. Through this convention, it would commit to provide equal economic, cultural, and political rights to men and women (Hadjipavlou and Mertan, 2019). International resolutions, conventions, and documents provide an important sanctioning framework for public institutions and nation-states to take steps toward gender equality. The Government of Northern Cyprus has made these commitments under United Nations and European Union documents. Another international convention on gender equality, the Council of Europe Convention on Preventing and Combating Violence against Women and Domestic Violence, was opened for signature in Istanbul on May 11, 2011 to combat violence against women and domestic violence. With the ratification of this Convention, called the Istanbul Convention for short, by the Republican Assembly of Northern Cyprus on December 5, 2011, the Assembly declared that it would take responsibility for preventing violence, protecting victims, and bringing perpetrators of violence to justice and that all members of society, especially men and boys, must change their attitudes (Mertan, 2000). Turkish Cypriot women in the northern part of Cyprus work outside the home, as is the case in other parts of the world. Although starting a business is good for women's self-confidence, financial independence and the opportunity to contribute to the production process can also bring some challenges. One of the biggest problems for working women is taking on domestic responsibilities at the same time. This burden is exacerbated, especially for working mothers. As a result, it becomes extremely difficult or sometimes impossible for women to participate in the social and political environment (Onuş, 2018). Based on the feminist perspective, human activities are divided into private and public spheres. For example, women are responsible for productive and domestic work such as housework and child-rearing, while men dominate public life, which includes a wide range of activities from paid work to politics. While the activities associated with women are seen as women's natural duties rather than work, those performed by men are seen as relatively more valuable. This has led to the exploitation of women's work in the private sector and their exclusion from the public sector, or even if they are represented there, they play a subordinate role (Tombak and Topdal, 2014). The exclusion of women from the public sphere, especially politics, underscores male domination in Northern Cyprus. Although women have gained equal rights with men in various fields, they have not been able to achieve leadership positions in the political structure of society (Yirmibeşoglu, 2008).

### 2.5 Current situation of women's participation in political life

Attempts have been made to actively shape the role of women in Northern Cyprus, but the number of female deputies in parliament is still lower than that of men. The number of female MPs is 11, while the number of male MPs is 39 (KKTC Cumhuriyet Meclisi, 2022). Northern Cyprus has had neither a female president nor a female prime minister. Following the assemblies of the Republic of Cyprus in 1960, the Turkish Federated State of Cyprus from 1975 to 1983, and the proclamation of the Turkish Republic of Northern Cyprus (TRNC) on November 15, 1983, there have been a total of 22 female deputies during the 50 years of these three periods. Since the establishment of the Turkish Republic of Northern Cyprus, two female deputies have served in the Ministry of the Assembly of the Republic. There was no female minister in the Council of Ministers formed in 2014. From 2018 to 2022, the Council of the Republican Assembly of Northern Cyprus could have only one female member. Another important debate in Northern Cyprus concerns the office of mayor in twenty-eight municipalities, which has been reduced to eighteen by 2022, but there is still no female mayor in Northern Cyprus. The number of male politicians in each election from 2014 to 2022 is higher than the number of female politicians for the membership of the municipality associated with the local administration. The number of male and female members of the municipality is one hundred and eighty-eight and ninety-two, respectively. The last important position is the mukhtar, which is the head of a village or district, and as with other political positions, the number of female mukhtars is less than the number of male mukhtars, with a large difference in numbers. From 2014 to 2022, the number of female and male heads is sixteen and two hundred and twenty-six, respectively. With the amendment of the Law on Political Parties in 2015, the 30% quota for nominated candidates came into force. Although the parties draw up the lists according to the legal regulations, this quota is not reflected in the election results (Tüccaroǧlu, 2012). From 1990 to 2013, there was not much difference in the number of female deputies. In 1990 and 1993, the number of female deputies was only two. In 1998 and 2003, the number increased to four female MPs. In 2005, however, there were only three. In 2009 and 2013, the number rose again to four female deputies. In 2018, it peaked with nine female politicians entering parliament. For many years, there was criticism that the number of female deputies in parliament was low. Even though the number of female deputies in the 50-seat Parliament of the Republican Assembly of Northern Cyprus in 2022 is eleven, it is still an unexpected figure (Orakcioǧlu, 2022).

## 3 Materials and methods

### 3.1 Research design and participants

The focus of this study was on qualitative research to gain in-depth knowledge and collect primary data on the political role of women in Northern Cyprus. To obtain open-ended responses from the participants and to capture their attitudes, feelings, and behaviors, semi-structured interviews were conducted with female members of the parliamentary executive bodies and assemblies of the three political parties, CTP, UBP, and HP, as well as with ministers of the Republican Assembly of Northern Cyprus. A total of twenty-one female politicians participated in the interviews. Eleven of them are female ministers in the Republican Assembly of Northern Cyprus as members of their parties CTP, UBP, and HP. Ten participants are female members of the central executive body and the National Assembly in CTP, UBP, and HP. Two participants are members of the party council of CTP and the other two participants are members of the central executive committee of the same party. While one participant is a member of the central executive committee of UBP, two participants are members of the party council of the same parties. While one participant is a member of the central executive committee of HP, two participants are members of the party council of the same party. Before starting the semi-structured interviews, it is important to note that the Ethics Committee of the European University of Lefke approved that the interview questions are impartial and formulated. Based on this approval, research data collection began on December 9, 2021, and ended on April 13, 2022. Due to the intensity of their work pace, participants were interviewed in person at a time and place convenient to them, and meetings were even held with some MPs in Parliament. The length of the interviews varied depending on the participants' responses. With the consent of the interviewees, a voice recorder was used and notes were taken to create a transcript. The data from the conducted interviews were categorized according to the questions collected under the headings related to the topic and analyzed by the researchers.

The demographics of the participants were included in the study based on their educational background and work history. One participant is a high school graduate, six participants have a doctoral degree, seven participants have a master's degree, and the other seven participants have undergraduate degrees. They come from different professional backgrounds. The professions of most of them, e.g., teachers or lecturers, doctors, and lawyers, belong to professional groups due to the reduction of the gender gap in education in Northern Cyprus. One participant is a civil servant in the field of agriculture and currently works in the Ministry of Agriculture in a managerial position. The agricultural field is mostly known as a male domain, but this suggests that working in these fields does not cause women to neglect or postpone their household duties, hinder their children's education, or disrupt daily family life. In February 2002, a law transformed the Department of Women and Family Affairs into the Department of Women's Studies. This law guarantees that women receive and improve their rights in terms of equality from the government (Lisaniler, 2006). Business owners are also generally perceived as men, but five participants run their businesses, both in their fields and started by their families. The other demographic information relates to the age of the participants. One participant is between 25 and 34 years old. Eight participants are between 35 and 44 years old and the other eight participants are between 45 and 54 years old. There are only four participants in the 55–64 age group. This shows that the participants are young women who can stand on their own feet after reaching a certain age after obtaining their profession. In terms of marital status, the majority of participants, sixteen, are married and have household responsibilities.

### 3.2 Data analysis

This study is based on a thematic analysis (Lochmiller, 2021) involving four steps: First, the entire transcription is reviewed to get an overall impression of what the female politicians said. The second step consists of rereading the statements to identify the units of meaning, taking into account the phenomena of interest. In this stage, common themes and codes were also identified and categorized by recurring key terms. In the third step, all meaning units are reviewed to gain deeper insight into the phenomena of interest, and in the final step, the transformed meaning units are synthesized into a consistent statement about the experiences of female politicians (Eysenbach and Köhler, 2002). The themes are distinct from each other and have the property of explaining the data by forming an inherently meaningful whole. The themes were chosen to be organized originally and not to contain the personal opinions of the authors while specifying the themes to increase the subjectivity of the study. Since it was decided to keep the identity of the participants anonymous and confidential for ethical reasons, the participants were coded in the transmission of the results as P1, P2,..., and P21 in the transmission of the results.

The purpose of this study is to examine why the country's traditional political culture keeps women out of the political arena and away from leadership positions, even though there is no visible gender discrimination in Northern Cyprus. This study is mainly about women's political participation, and it aims to show why female representatives still cannot reach leadership positions in politics, although it is not difficult for women to participate in social life in Northern Cyprus. This article is useful and interesting, especially given the challenges women face and their underrepresented status in the political sphere. This study contributes to the literature and policy by providing new empirical evidence of gender inequality in politics in the context of Turkish Cypriot women politicians. It also critically and comprehensively examines the difficulties, struggles, and experiences of women politicians living on the island as they attempt to enter politics. One of the limitations of the study is that male politicians could also be included in the survey to provide more comprehensive information and compare the responses of female representatives. However, the study intends to be based on the opinions of female politicians. Considering the data collection method and reliability of coding, there is another limitation of the study. Reliability is related to the repeatability of research results and to the question of if the study can be conducted once again after the first one, and whether it would yield the same results. The consistency with coding segments placed in the same category is referred to as reliability. In addition, the reliability of the coding criterion means that the findings and interpretations of the research are the product of a consistent process by which the findings obtained would be as clear and reproducible as possible (Compton et al., 2012). This study contains controversial questions due to the semi-structured data collection method. This has led to the collection of subjective data that is prone to bias. Even if the same work is repeated with the same people, the responses will vary because there are questions that can be raised for discussion. At the same time, there are two types of reliability of coding: inter-coder reliability refers to consistency between different coders, while intercoder reliability refers to consistency in how the single researcher codes data at multiple points (O'Connor and Joffe, 2020). In this study, intra-coder reliability is used that represents code entered by a single coder and this reduces the reliability of the study. Since the analysis may need some subjective judgment to be shared between researchers. As a recommendation and for further studies, the inter-coder reliability can be endeavored for other relevant cases to contribute to the literature.

## 4 Results and discussions

### 4.1 The reasons for difficulties faced by women politicians

The interview questions are divided into two groups, which are asked to the participants using the semi-structured interview technique. The reasons for the difficulties faced by female politicians in participating in politics and the possible solutions proposed are the focus of consideration of the content of the questions. However, since focusing on the entire set of questions is beyond the scope of this article, it will mainly focus on the causes of the problems faced by female MPs when participating in politics. Among the questions in the first group, one of the most important questions, in particular, is “What are the opportunities and challenges for women's political participation in Northern Cyprus?” As shown in Table 1, there are five codes under the theme of “opportunities”: “self-improvement,” “self-confidence,” “modern structure of our society,” “access to education and economic independence,” and “gender quota.” One participant elaborates on the self-improvement of individuals through participation in politics. She (P1) asserts: “*Self-development is an opportunity by taking on a social duty”*. Based on her statement, women's desire to improve themselves is an opportunity. Women and men are at the same level of self-development in Northern Cyprus. Women can make their own educational and professional decisions just like men. In other words, they have the opportunity and access to education to better themselves. Self-development is an opportunity to take on a social task and improve oneself in certain subjects, in a profession, or career. This means that women and men in North Cyprus are at the same level of self-development. The same participant also addresses women's self-confidence as an opportunity. She points out women's domestic duties, but this can be seen as an opportunity, as it represents women's ability to manage work, domestic duties, and politics together. Women's participation in politics and fulfilling domestic duties at the same time can cause anxiety, but this can also be an opportunity because it shows that women can manage all these tasks and increase their self-confidence. The codes “modern structure of Turkish Cypriot society” and “access to education and economic independence” show that women and men in Northern Cyprus have equal opportunities when it comes to participating in education and employment. Three participants identify respectively: “*No one can say to the person who will join the politics you cannot join”* (P6), “*Women can have professional life by support of their family members for their domestic responsibilities”* (P9), and “*The social and cultural structure of Turkish Cypriot society as an opportunity and this structure does not cause any problems to women”* (P12). According to these participants, young girls or women can participate in educational life and achieve their economic freedom without any problems, just like men. Family members are supportive due to the democratic family structure in Northern Cyprus. Women's access to educational and business life is also quite good, which enables women to participate more in public life. The democratic structure of the Turkish Cypriot community does not make it difficult for young girls and women to participate in public life. No one tells someone who wants to be involved professionally or politically that they cannot. Young girls and women have the freedom to decide on their profession or get involved politically. Finally, most respondents (eleven participants) agree with the idea that gender quotas are the most important and only opportunity for women in North Cyprus. Gender quotas have been instrumental in increasing the number of women in politics and in the decision-making mechanisms of political parties, local councils, and parliaments. The higher number of women can lead to greater representation of women and give them more acceptance in society. Increasing the number of women in politics can also help promote other women in society. Participants also emphasize that gender quotas are not intended to meet quotas, but to raise society's awareness that women should participate in politics with their thoughts, principles, and attitudes.

**Table 1:** Existing opportunities and challenges for women's political participation.

**Thema**	**Code**	***f***
Opportunities	Improving self	1
	Self confidence	1
	Modern structure of our society	3
	Access to education and economic independence	3
	Gender quota	11
Challenges	Lack of encouragement by their political parties	4
	No opportunity for women political participation in TRNC	4
	Traditional state perspective and male dominance	8
	Domestic responsibilities	14

In the second part of the question, participants mainly indicate that the challenges to women's participation in politics are greater than their opportunities. There are four codes related to “challenges” as indicated in Table 1. These are “lack of encouragement from political parties,” “no opportunity for women's political participation in North Cyprus,” “traditional state perspective and male dominance” and “family and work commitments.” The first two codes, “lack of encouragement” and “no opportunity for women's political participation in Northern Cyprus,” originate from the political difficulties faced by women representatives, both within their parties and in government in general. According to the participants, the active position of women in political parties should be strengthened first and foremost, and girls should be educated in political parties from a young age to gain knowledge about politics. Women should not only be used to fill gender quotas in the two political parties or other political bodies of Northern Cyprus. The third code is that the government in Northern Cyprus has a traditional state perspective, namely a patriarchal structure that makes it difficult for women to participate in politics. Turkish Cypriot society lives in a democratic country, but in the practice of the democratic government of Northern Cyprus, the representation of women in politics is not easy which shows there is a male dominance structure in the participatory decision-making mechanisms in politics. This is also due to the aforementioned separation of public and private spheres in society. The roles that women occupy in society, such as domestic responsibilities, make it difficult for them to be more involved in politics. Therefore, the male-dominated political structure leads to the exclusion of women in this environment. Participants also cite that the public's expectations of a woman entering politics are higher than a man, for example, because more qualifications are required. There is prejudice against women being able to be active in politics as men, and women are expected to be more educated and qualified than their male counterparts. The participants are primarily convinced that this problem is due to the traditional political culture in Northern Cyprus, as shown in the fourth code. Although women have the same rights as men in many areas, they do not have the same authoritarian status as men in the political structure of society. The sociocultural structure of their society prevents women from actively participating in political life. Political discussions are also held in male-dominated areas such as village cafés or sports clubs, and women have always assumed the role of wife or mother in their homes.

In the context of male dominance in the political structure, the study of Erbilen et al. (2021) focuses mainly on the dilemma between the private and public spheres, which has been at the center of two centuries of feminist literature and political struggle. The dichotomy of public and private is often used to construct, control, limit, exclude, and oppress gender and sexual differences to preserve traditional patriarchal power structures. This dichotomy also forces both genders to assume certain roles within the patriarchally defined boundaries of the public sphere. Although women emerge as important actors in social life, they are rarely considered political leaders. The main problem with this distinction is that while women have the same rights as men in many areas, they do not have the same authoritarian status as men in the political or economic structures of society. Although they were allowed to participate in the Republican Assembly in Northern Cyprus, they have made little progress in gaining real political power in many countries because participants support the notion that there is a male-dominated structure to politics in participatory decision-making mechanisms. When women's household and childcare responsibilities are added, women's representation in politics becomes more difficult (Bedioǧlu and Batman, 2014; Maguire, 2018). Some participants specify in their statement: “*We are living in a democratic country. but when it comes to practice in terms of representation of women in politics, women face difficulties in decision-making mechanisms due to traditional male dominance structure in society and in politics”* (P9), “*Women's home, family, and child responsibilities are main difficulties that need more effort. the state has nothing to do with this situation”* (P12), and “*If women have family responsibilities, it is much more difficult to be active in politics because it takes time and there is no private life. This is not a problem for men but it is for women because women undertake these responsibilities due to male-dominant structure”* (P16). Politics is therefore seen as a male preserve, and even when women do vote, they inevitably make their electoral decisions along the lines of their fathers, husbands, and brothers. The consideration that the places where politics is made are suitable for men entails the idea that politics is men's business. Because of the dilemma between the private and public spheres, women are constrained by domestic duties. They are expected to marry, fulfill their domestic duties, and raise their children. With these duties, women are removed from the public sphere (Fraser, 1990).

As mentioned in the study by Erbilen et al. and Seltzer et al. (1997) also point out the difficulties for women who want to participate in politics. They address the male-dominated state perspective and women's domestic responsibilities. Based on the male-dominated state perspective, women should work harder to create a negative perception in the media or to convince those who do not want to vote for them. In Northern Cyprus, however, sports clubs, pubs, and coffee houses are the preferred venues for direct contact with voters. These places cause difficulties for both voters and women candidates, as propaganda activities in these places are carried out either in the evenings or on weekends. The physical inadequacies of the preferred places and the male perspective of the users of the space are effective. Based on the feminist perspective, the division of the public and private spheres in society can also cause this difficulty (Altindal, 2009). The participants indicate that the traditional division of labor between men and women has an impact on women's political life. In the private sphere, domestic duties are women's tasks, and managing the house is men's task. The social division of labor keeps women trapped in the private sphere and pushes them into a passive position. It leads to the man becoming the subject in the public sphere. Even when women are politically engaged in the public sphere, they still bear the responsibility for their homes. Looking at the political history of the country, it is clear that women are very rarely represented in the political scene as mukhtars, mayors, party leaders, and members of parliament, and are not perceived as doing only men's work.

One of the most important questions is about the role of women in key executive and local government positions in Northern Cyprus. The first part of the question discusses the reasons why women do not occupy key leadership positions in political parties, although there are women in parliament. As shown in Table 2, the topic of the first part of the question is “reasons for the absence of women in key leadership positions” and the code is “male dominance in key leadership positions.” Participants emphasize that the number of female MPs increased from nine to eleven in the last election in 2022 in Northern Cyprus. This is a welcome situation, even if the desired number is not reached. However, even though women are represented in parliament, it is still difficult for women to obtain important leadership positions in political parties. All participants agree that this is due to the patriarchal structure (male dominance) of society and politics in Northern Cyprus. The second part of the question is about why there is no female mayor in local government in North Cyprus. All participants agree that the lack of female representatives in the local government is a major problem because women have more detailed-thinking personalities than men. As mentioned in Holman's (2017) study, women can be more successful in key leadership positions and local governments. Participants explain this by the recognition of politics as a man's job, the masculine structure of the places where political work is done, the traditional division of labor between women and men in the Turkish Cypriot community, and the greater commitment of women to politics. Although women and men seem to have equal rights in society, the number of female local representatives in Northern Cyprus is very low compared to men, and to date, there has been no female mayor.

**Table 2:** Key executive and local government positions of women in TRNC.

**Thema**	**Code**	***f***
Reasons for lack of women in key executive positions	Key executive positions male dominance	21
Reasons for no female mayor in local governments	Local government male dominance	21

### 4.2 Impact of traditional values and social/cultural norms on political participation of women

The impact of traditional values played an important role in discussing the difficulties women face in participating in politics. For this reason, participants were asked the question, “Do traditional customs, social and cultural norms affect the role of women in politics?” As can be seen in Table 3, culture and traditional values have a great influence on the role of women politicians. According to the participants' responses, the theme is: “Impact of cultural norms on women's political participation.” Only one participant believes that cultural norms are decreasing. According to this participant, traditional norms are decreasing compared to the past, and taboos about the role of women in society are gradually being broken. Young girls and women today can do what they want more easily than in the past. However, the remaining majority of participants agree with the great influence of traditional values and cultural customs on politics. As also mentioned in Thanikodi and Sugirtha's (2007) study, women's political participation is limited due to traditional values. Domestic responsibilities are placed on women's shoulders, making it difficult for them to participate in politics. Therefore, most participants emphasize that the patriarchal structure of society in Northern Cyprus hinders women's political role. Some of them argue: “*There are conservative values in political parties due to male-dominated structure… According to the male-dominated political structure, domestic responsibilities are accepted as a main obstacle for women to be elected as a mayor”* (P3), “*Turkish Cypriot Community has a male hegemony in politics and this does not allow women to have managerial positions… I believe women are more successful in local governments due to their detailed thinking structure but male dominance political structure hinders this”* (P6), and “*Women do not have key executive positions in political parties and local governments due to the male-dominance structure of politics”* (P10). To emphasize the masculine structure of politics, they point to the acceptance of politics as men's work and the places where political work is done, such as coffee houses, sports clubs, or taverns. The consideration that these places are suitable for men brings the idea that politics is men's work. Because parties are male-dominated, women are often excluded from men's decision-making processes.

**Table 3:** Impacts of traditional customs, social and cultural norms on the role of women in politics.

**Thema**	**Code**	***f***
Impacts of cultural norms on political participation of women	Decreasing the impacts of cultural norms	1
	Cultural norms affect the role of women	20

One of the most crucial questions to capture society's image of women as politicians is whether or not they feel social pressure. As shown in Table 4, nine participants respond “yes, social pressure” on the topic of social pressure as a female politician. They state that they feel pressure because of the patriarchal structure of society. The roles assigned to women, such as domestic duties, mean that they do not have time for politics. Similar to the study by Paxton et al. (2007), participants show that politics is a male profession and women cannot adequately represent the public like men. The participants relate this issue to the patriarchal structure of society to explain the pressure they feel. The remaining twelve female participants confirm that they do not feel social pressure. In their opinion, the reason they do not feel pressure may be that they live in a democratic family and country or that they are free to work in the same way as men.

**Table 4:** Feeling social pressure as a female politician.

**Thema**	**Code**	***f***
Feeling social pressure as a female politician	Yes	9
	No	12

The next question refers to whether the participants have politicians in their family members to discuss the traditional structure of politics in Northern Cyprus. As shown in Table 5, one participant did not indicate whether she has a politician in her family. Ten participants indicated that they have a politician in their family, and the remaining ten people do not. The second part of the question asks whether the presence of a politician in the family increases the visibility of women. There are three codes under the theme “the role of family members of politicians on women's political participation”. These codes are: “visibility in the family depends on the individual,” “biases about skills come from family members,” and “easier visibility for women who have politicians in their family.” Four participating representatives emphasize that women's visibility depends on the individual. According to these four participants, women may have politicians in their families, but that does not mean they will be visible and successful no matter what. Being visible, they argue, is related to the person. The person should be visible in politics because it is related to their education, knowledge, attitude, and public appearance. The next code is “Prejudices about abilities come from family members.” While two participants mention the code “family visibility depends on the person,” they also emphasize that this situation can lead to prejudice against women who are politicians among their family members. Participants state that if there are women politicians in their family, they may have evolved because of their family members, but at the same time, this may lead to prejudice that they can be more easily recognized because of their family members. Participants disagree with this situation. If people have attained a position, it is because of their efforts and abilities, not because of their family members. Even the participants who argue that getting to a place because of family ties does not bring lasting success point out that this prejudice is unfounded. Finally, according to most participants, recognition and visibility are easier when one of their family members is a politician. These participants indicate that this is beneficial for both recognition and being a role model in politics at home. Thus, a new member who comes from a family with a political background has more political experience. In a small community, it is easier for people who have politicians in their family to make their mark because people know each other best.

**Table 5:** Having politician family members.

**Thema**	**Code**	***f***
Having politician family members	No information about having politician family member	1
	Have politician family member	10
	Have not politician family member	10
Role of politician family members on women political participation	Family visibility depends on the individual	4
	Prejudices about capacities come from family members	4
	Easier visibility for women having politicians in their families	15

### 4.3 Improvement of the position of women in politics

So far, the study has discussed the perceived reason for women's participation in politics in Northern Cyprus based on their views and thoughts of them in interviews. To conclude, the participants were also asked about possible solutions to the problems of women in politics. One of the most important questions is: “How can the role/position of women in politics be improved? What have you personally done to achieve this?” As can be seen in Table 6, the theme for the first part of the question is “ways to improve the role of women in politics,” based on participants' responses. There are five codes for this theme: “the party's attitude improves the role of women in politics,” “support from society,” “laws on women's rights should be improved,” “self-confidence,” and “women should be motivated in politics.” One participant emphasizes the importance of the party's attitude toward the role/position of women in politics. If the necessary arrangements are made within the party, the role of women in politics will be strengthened so that women's chances of being elected can increase. She also points to the importance of gender quotas, which can increase the number of women in political parties, which in turn can encourage other women to become involved in political parties as well. The other participant emphasizes public support to improve the role of women in politics, but this is not enough, and women in general need to be properly motivated to be active in politics. If society encourages women and wants to see more women in politics, women may become more enthusiastic about politics. As mentioned in the study by Hooks (1986), the participant in question points out the importance of society's solidarity against male domination. With solidarity, any problem that affects women in society can be addressed more sensitively. This will also help to strengthen women's power and they can overcome problems more easily. The other three participants believe that the role of women in politics can be strengthened by passing laws on women's rights. The state should create new legal regulations to facilitate women's social, economic, and political life. With these laws, women's position in society and politics can be strengthened, and women can increase their positions in politics in the long run. Participants assert: “*Executive authorities, ministers, etc. should be trained to prepare efficient laws for women's rights”* (P4) and “*To increase political participation of women, more laws related to women or social issues should be passed”* (P14). According to the participants, women's rights can be protected through various laws, and women can prove themselves in politics and increase their credibility by appearing less vulnerable in the eyes of society. In addition, women can be very independent and take care of their family members, but they may face problems at certain times due to their household duties. If the necessary laws are enacted, the problems against women can also be ended and women can consolidate their place in politics. For example, the state should provide a kindergarten for women with children and nursing homes to care for the elderly. The necessary legal regulations should be made to solve the problems of women with such tasks (Dubler, 2003).

**Table 6:** Ways to overcome the challenges to women's political participation.

**Thema**	**Code**	***f***
Ways to improve role or position of women in politics	Attitude of party improves women's role in politics	1
	Support from society	1
	Laws on women's rights should be increase	3
	Self confidence	7
	Women should be motivated in politics	11
Things have been done personally	Did nothing personally	2
	Personally encouraged women to improve women's role in politics	7
	Personally attended to gender training and activities to encourage other people	13

As shown in Table 6, seven participants mentioned the importance of having “self-confidence” to prove themselves in politics. However, from a feminist perspective, a male-dominated society has weakened women's self-confidence. Self-confidence, which is seen as a promise of liberation, is essential for feminist political empowerment because it conveys the courage to stand up to oppressive structures and change them. The feminist concept of self-awareness consists of the idea of respect for the individual “I” (Dillon, 1992). Therefore, participants believe that if a woman continues to improve herself in all circumstances, no matter what her profession, that success will continue when she enters politics, and that this may lead her to perform more easily in politics. For example, women are in many different professions, such as surgeon, property manager, or engineer, meaning women can succeed in all areas of society in terms of education and careers. Politics is also one of the areas in which women can be successful. Political parties can pave the way for women by providing them with a suitable environment for decision-making mechanisms to boost their self-confidence. This can also prevent women from lagging behind men. Finally, on the topic of “ways to improve the role or position of women in politics,” the code formulated by eleven participants is “women should be motivated in politics.” Some of the participants specify: “*As women in the party, we are constantly fighting for the establishment of various mechanisms within the party”* (P3), “*Political parties should organize different events for women to increase awareness on the role of women in political”* (P8), “*Women's organizations in parties should be raised. Men and women should be programmed the same”* (P12), and “*Hardworking and social awareness in political parties can improve the role of women in politics… Struggles in political parties for the development of women's freedom can increase their position in politics”* (P16). According to most participants, women need to be motivated to be more active in politics. Women should be encouraged to join a political party at a young age to learn about political structures and gain experience in politics. They also stress the importance of raising public awareness through training on gender issues. Educating people about gender awareness at a young age can develop a mindset based on gender sensitivity that can change perspectives. Because the only way to give shape to cultural values is through public education and awareness. Participants mainly agree our society needs gender studies for both genders men and women together. Gender studies can bring up many issues related to women's difficult situations or problems to increase awareness. They also argue education should be started from a young age because the mentality of the male-dominated political system is only changed by education. If children can have awareness and sensitivity about disadvantaged groups of people, especially women, from a young age, the risk of society's gender inequalities can be reduced (Atchison, 2016).

The second part of the question asks participants about the things they personally do to improve the role or position of women in politics. As can be seen in Table 6, the theme is “things done personally” based on the participants' responses. There are three codes for this theme, namely: “have personally done nothing,” “have personally encouraged women to improve the role of women in politics,” and “have personally participated in gender training and activities to encourage others.” Seven participants emphasized encouraging women to participate in politics above all else. They have tried to verbally encourage women to participate in politics. This is because when women in politics share their experiences with other women, they can be encouraged and accelerate their participation in politics. Finally, the majority of participants personally encourage other people, especially women, to participate in gender education and activities. According to these participants, it is easier to set an example for future generations who have more experience with women's rights or the importance of women in politics. They make great efforts to raise awareness in their respective political parties by organizing various activities about gender studies and the development of women's role in politics. They also focus on the importance of public awareness, which can be raised through projects, seminars, or especially training on gender awareness. The mentality of society regarding gender roles needs to change to increase the visibility of women in the public sphere. Above all, participants emphasize the importance of educating children about gender equality at a young age. In the school system, boys are still encouraged to play soccer and girls with dolls. In their opinion, it is very difficult to solve this problem with this type of education system. According to the participants, it is easier to set an example for future generations who have more experience with women's rights or the importance of women in politics. They try very hard to raise awareness about their respective political parties by organizing them differently.

## 5 Conclusion

The main purpose of this article is to highlight the low representation of women in the political arena in Northern Cyprus and to examine the main limitations of Turkish Cypriot female representatives. Based on semi-structured interviews, the views of female MPs on the impact of gender inequality on their political life were explored by overcoming all factors that exclude them from politics. The patriarchal system was highlighted to explain the power interactions between men and women based on the feminist perspective. Women were kept out of public life by maintaining the public and private spheres. In general, it was found that there are various limitations due to the specific conditions in Northern Cyprus. The result of this research is that traditional values related to gender are the biggest obstacles to women's political participation. Women who want to actively participate in politics are faced with certain expectations that are never required of male candidates. First of all, a woman is required to prove herself to all voters in terms of education, social status, and economic income level. Based on the public and private dilemma, the pressure to manage domestic duties and politics together leads to problems for women. In politics, there are no fixed working hours, such as participating in propaganda activities, traveling, and lengthy meetings. Therefore, women have little time for their personal lives, and they rely on the support of other women in their immediate environment. If they feel they do not receive adequate support, they stay away from active politics and wait until their children are grown to relinquish some of their responsibilities. The times and places where politics is done are also not suitable for women and pose problems for them. In this context, women try to exist as assistants who address male politicians instead of making policies themselves. Women need to free themselves from these positions and meet in an environment where they can share their ideas about the country's problems, engage in qualified active politics, and conduct self-confident and awareness-raising activities. These meetings will motivate women to participate in politics. Women's participation in politics is absolutely necessary to change traditional gender roles and make women more visible in the public sphere. In this way, it will be easy to speak of a true democracy in which all parts of society are equally represented.

## Data availability statement

The original contributions presented in the study are included in the article/supplementary material, further inquiries can be directed to the corresponding author.

## Ethics statement

The studies involving humans were approved by European University of Lefke Ethics Committee with the number ÜEK/65/01/09/2122/01. The studies were conducted in accordance with the local legislation and institutional requirements. The participants provided their written informed consent to participate in this study. Written informed consent was obtained from the individual(s) for the publication of any potentially identifiable images or data included in this article.

## Author contributions

NA: Writing – original draft. EO: Writing – original draft.

## References

[B1] Altindal Y. (2009). Erkeksi siyasetin erk'siz dublörleri. Balikesir Üniversitesi SBE Dergisi 12, 351–367.

[B2] Atchison A. L. (2016). Bringing women in gender mainstreaming in introduction to political science. Polit. Sci. Polit. 49, 546–549. 10.1017/S1049096516000950

[B3] Bedioǧlu M. Batman K. A. (2014). University students' perception on women's gender role: a case of North Cyprus. Kadin/Woman, 2000. J. Womens Stud. 15, 45–71.

[B4] Chin J. L. Lott B. Rice J. K. Sanchez-Hucles J. (eds.). (2007). Women and Leadership: Transforming Visions and Diverse Voices. Blackwell Publishing.

[B5] Compton D. Love T. P. Sell J. (2012). Developing and assessing intercoder reliability in studies of interaction. Sociol. Methodol. 42, 348–364. 10.1177/0081175012444860

[B6] De Nicola B. (2017). “Women and politics from the steppes to world empire,” in Women in Mongol Iran: The Khatuns (Edinburg, TX: Edinburgh University Press), 34–36.

[B7] Dillon R. S. (1992). Toward a feminist conception of self-respect. Hypatia 7, 52–69. 10.1111/j.1527-2001.1992.tb00697.x

[B8] Dubler A. R. (2003). In the shadow of marriage: single women and the legal construction of the family and the state. Yale Law J. 112, 1641–1715. 10.2307/3657498

[B9] Duner B. (1999). Cyprus: North is North, and South is South. Sec. Dialog. 30, 485–496. 10.1177/0967010699030004009

[B10] Erbilen S. Ü. Sabanci T. ve Kavci T. A. (2021). Kuzey Kibris Türk Cumhuriyeti, Cumhuriyet Meclisi'nde kadin Olmak. Insan ve Toplum Bilimleri Araştirmalari Dergisi 10, 2373–2395 10.15869/itobiad.885648

[B11] Eysenbach G. Köhler C. (2002). How do consumers search for and appraise health information on the World Wide Web? Qualitative study using focus groups, usability tests, and in-depth interviews. BMJ 9, 573–577. 10.1136/bmj.324.7337.573 11884321 PMC78994

[B12] Franceschet S. Krook M. L. Piscopo J. (eds.). (2012). The Impact of Gender Quotas. Oxford: Oxford University Press.

[B13] Fraser N. (1990). Rethinking the public sphere: a contribution to the critique of actually existing democracy. Soc. Text 25, 56–80. 10.2307/466240

[B14] Freedman E. B. (2002). No Turning Back: The History of Feminism and the Future of Women. New York, MY: The Random House Publishing Group.

[B15] Galligan Y. (2006). Bringing women in global strategies for gender parity in political representation. Univ. Maryl. Law J. Race Rel. Gender Class 6, 320–336.

[B16] Hadjipavlou M. Mertan B. (2010). Cypriot feminism: an opportunity to challenge gender inequalities and promote women's rights and a different voice. Cyp. Rev. 22, 247–268. Available online at: https://cyprusreview.org/index.php/cr/article/view/212

[B17] Hadjipavlou M. Mertan B. (2019). A multilevel intervention: the case of the Cyprus gender advisory team (GAT) achievements and challenges. J. Peacebuild. Dev. 14, 1–13. 10.1177/1542316619843258

[B18] Holman M. R. (2017). Women in local government: what we know and where we go from here. State Local Gov. Rev. 49, 285–296. 10.1177/0160323X17732608

[B19] Holmes B. (2022). Citizens' Engagement in Policymaking and the Design of Public Services. Research Paper, 1, 2011–12. Available online at: https://parlinfo.aph.gov.au/parlInfo/download/library/prspub/942018/upload_binary/942018.pdf;fileType=application/pdf#search=%222010s%22 (accessed March 15, 2022).

[B20] Hooks B. (1986). Sisterhood: political solidarity between women. Fem. Rev. 23, 125–138. 10.1057/fr.1986.25

[B21] Hooks B. (2000). Feminism is for Everybody Passionate Politics. London: Pluto Press.

[B22] Hughes M. M. Paxton P. Clayton A. B. Zetterberg P. (2019). Global gender quota adoption, implementation, and reform. Comp. Polit. 51, 219–238. 10.5129/001041519X15647434969795 PMC10978000 38549789

[B23] Hunt S. (2007). Let women rule. For. Aff. 86, 109–120. Available online at: www.foreignaffairs.com/articles/2007-05-01/let-women-rule

[B24] Kenworthy L. Malami M. (1999). Gender inequality in political representation: a worldwide comparative analysis. Soc. Forces 78, 235–268. 10.2307/3005796

[B25] KKTC Cumhuriyet Meclisi (2022). Milletvekilleri. Available online at: http://www.cm.gov.nc.tr/Milletvekillerimiz1 (accessed June 25, 2023).

[B26] Kramer A. Gehred K. Kirillova L. Duguid B. Weber S. Holmes M. S. (2019). The Feminism Book: The Big Ideas Simply Explained. New York, NY: Dorling Kindersley Limited.

[B27] Lisaniler G. F. (2006). Gender equality in North Cyprus (Turkish Republic of Northern Cyprus). Cyprus: The Mediterranean Mallinson, 133–140.

[B28] Lochmiller C. R. (2021). Conducting thematic analysis with qualitative data. Qual. Rep. 26, 2029–2044. 10.46743/2160-3715/2021.5008

[B29] Maguire S. (2018). Barriers to Women Entering Parliament and Local Government. IPR Report. Available online at: https://www.bath.ac.uk/publications/barriers-to-women-entering-parliament-and-local-government/attachments/barriers-to-women.pdf (accessed May 21, 2022).

[B30] Mallinson W. (2009). Cyprus: A Historical Overview. Republic of Cyprus: Press and Information Office.

[B31] Masad R. (2020). The Struggle for Women in Politics Continues. Available online at: https://www.undp.org/blog/struggle-women-politics-continues (accessed June 25, 2023).

[B32] Mertan B. (2000). Doǧu Akdeniz Üniversitesi'nde kadinin konumu: Bir inceleme. J. Women Stud. 1, 1–9. 10.22559/folklor.1849

[B33] Mindzie M. A. (2015). “Enhancing the political participation of women and youth,” in Building Peace and Development in the Sahel: Enhancing the Political Participation of Women and Youth. International Peace Institute. Available online at: http://www.jstor.org/stable/resrep09525.7 (accessed April 15, 2022).

[B34] O'Brien D. Z. Rickne J. (2016). Gender quotas and women's political leadership. Am. Polit. Sci. Rev. 110, 112–126. 10.1017/S0003055415000611

[B35] O'Connor C. Joffe H. (2020). Intercoder reliability in qualitative research: debates and practical guidelines. Int. J. Qual. Methods 19, 1–13. 10.1177/1609406919899220

[B36] Onuş E. (2018). Kibris Türk Varoluş Mücadelesi Konulu Eserlerde Kadin Kahramanligi. J. Turk. Lang. Literat. 4, 206–228. 10.20322/littera.355519

[B37] Orakcioǧlu E. (2022). Meclisin *kadin Vekil Sayisi 11. Kibris Gazetesi*. Available online at: https://www.kibrisgazetesi.com/kibris/meclisin-kadin-vekil-sayisi-11-h124143.html (accessed January 25, 2022).

[B38] Paxton P. Kunovich S. Hughes M. M. (2007). Gender in politics. Annu. Rev. Sociol. 33, 263–284. 10.1146/annurev.soc.33.040406.131651

[B39] Schrupp A. (2017). A Brief History of Feminism Translated by Sophie Lewis. The Massachusetts Institute of Technology Press.

[B40] Seltzer R. A. Newman J. Leighton M. V. (1997). Sex as a Political Variable-Women as Candidates and Voters in U.S. elections. Boulder, CO: Lynne Reinner.

[B41] Solsten E. (1993). Cyprus: A Country Study. Washington, DC: Federal Research Division Library of Congress.

[B42] Thanikodi A. Sugirtha M. (2007). Status of women in politics. Ind. J. Polit. Sci. 68, 589–606.

[B43] Tombak A. Topdal E. B. (2014). Kuşaklar boyu kadin olmak. Yaratici Drama Dergisi 9, 60–67. 10.21612/yader.2014.006

[B44] Tong R. (2009). Feminist Thought: A More Comprehensive Introduction. Boulder, CO: Westview Press.

[B45] Tüccaroǧlu I. (2012). Kuzey Kibris Türk Cumhuriyeti'nde Kadinin Siyasal Yaşamda Karşilaştigi Güçlükler. Kadin Araştirmalari Dergisi, 0/6. Available online at: https://dergipark.org.tr/tr/pub/iukad/issue/734/7944 (accessed July 10, 2023).

[B46] Vatandaş C. (2007). Toplumsal Cinsiyet ve Cinsiyet Rollerinin Algilanişi. J. Econ. Cult. Soc. 35, 29–56.

[B47] Wischermann U. (2004). Feminist theories on the separation of the private and public: looking back, looking forward. Women German Yearb. 20, 184–197. 10.1353/wgy.2004.0011

[B48] Yirmibeşoglu G. (2008). Constraints on women politicians in Northern Cyprus. Equal Opport. Int. 27, 692–708 10.1108/02610150810916758

[B49] Yukl G. (2006). Leadership in Organizations, 6th Edn. Upper Saddle River, NJ: Pearson-Prentice Hall.

